# Understanding the implementation and adoption of a technological intervention to improve medication safety in primary care: a realist evaluation

**DOI:** 10.1186/s12913-017-2131-5

**Published:** 2017-03-14

**Authors:** Mark Jeffries, Denham L. Phipps, Rachel L. Howard, Anthony J. Avery, Sarah Rodgers, Darren M. Ashcroft

**Affiliations:** 1 Centre for Pharmacoepidemiology and Drug Safety, School of Health Sciences, University of Manchester, Manchester, UK; 2 Research Pharmacist, Isle of Wight, UK; 3 Division of Primary Care, University of Nottingham, Nottingham, UK; 4 NIHR Greater Manchester Primary Care Patient Safety Translational Research Centre, University of Manchester, Manchester Academic Health Sciences Centre (MAHSC), Manchester, UK

**Keywords:** Medication safety, Prescribing, Information technology, Realist evaluation, Sociotechnical, Primary care

## Abstract

**Background:**

Monitoring for potentially hazardous prescribing is increasingly important to improve medication safety. Healthcare information technology can be used to achieve this aim, for example by providing access to prescribing data through surveillance of patients’ electronic health records. The aim of our study was to examine the implementation and adoption of an electronic medicines optimisation system that was intended to facilitate clinical audit in primary care by identifying patients at risk of an adverse drug event. We adopted a sociotechnical approach that focuses on how complex social, organisational and institutional factors may impact upon the use of technology within work settings.

**Methods:**

We undertook a qualitative realist evaluation of the use of an electronic medicines optimisation system in one Clinical Commissioning Group in England. Five semi-structured interviews, four focus groups and one observation were conducted with a range of stakeholders. Consistent with a realist evaluation methodology, the analysis focused on exploring the links between context, mechanism and outcome to explain the ways the intervention might work, for whom and in what circumstances.

**Results:**

Using the electronic medicines optimisation system could lead to a number of improved patient safety outcomes including pre-emptively reviewing patients at risk of adverse drug events. The effective use of the system depended upon engagement with the system, the flow of information between different health professionals centrally placed at the Clinical Commissioning Group and those locally placed at individual general practices, and upon variably adapting work practices to facilitate the use of the system. The use of the system was undermined by perceptions of ownership, lack of access, and lack of knowledge and awareness.

**Conclusions:**

The use of an electronic medicines optimisation system may improve medication safety in primary care settings by identifying those patients at risk of an adverse drug event. To fully realise the potential benefits for medication safety there needs to be better utilisation across primary care and with a wider range of stakeholders. Engaging with all potential stakeholders and users prior to implementation of such systems might allay perceptions that the system is owned centrally and increase knowledge of the potential benefits.

## Background

Recent studies examining prescribing of medicines in primary care have highlighted the risks associated with this activity [1]. For example, one study estimated that approximately 13% of patients have experienced an adverse drug event (ADE) after receiving prescription medication in primary care and that many are serious enough to require hospital attention [2]. In addition, an estimated 8–12% of all hospital admissions are caused by ADEs, of which around 50% are preventable [2, 3]. Not only is there an increasing volume of prescribing in primary care (currently over 1 billion prescription items per year in England alone) [4], but increasing numbers of patients with multi-morbidities have led to a greater prevalence of polypharmacy [5], increasing the likelihood of an ADE [6, 7]. This makes the monitoring of medication use increasingly important for primary care patient safety.

Healthcare information technology (IT) can offer potential benefits for medication safety [8] for instance by providing easier access to prescribing data, facilitating clinicians’ assessments of the quality and safety of prescribing [1, 9, 10]. Lainer and colleagues [11] systematically reviewed randomized controlled trials of IT interventions including computerized physician order entry, clinical decision support systems and pharmacy information management systems. They concluded that these interventions successfully reduced medication errors but only for a limited number of clearly defined errors. Clearly, IT needs careful implementation to avoid its effectiveness being impaired by human factors such as alert fatigue and user inexperience [12]. Within primary care, the implementation of clinical decision support systems has been reported to lack compatibility with the general practitioner’s (GP) pre-existing work practices because these systems were found to correct decisions retrospectively, rather than provide guidance beforehand [13]. Meanwhile, the PINCER trial [14] found that an IT-based intervention to identify and correct medication errors in general practice was more effective when combined with dedicated support from a clinical pharmacist; the latter acting as a “change agent” who built working relationships with the practice staff [15].

From a sociotechnical perspective healthcare interventions involving the implementation of IT may be understood as complex interactions and interdependencies between the working practices of people using the technology, the organisational and social context, and the technology itself [16–20]. Such an approach takes into account the complex nature of healthcare and the organisational aspects of the workplaces in which interventions are implemented [13]. The current study examines the implementation of an electronic medicines optimisation system, (EMOS) that was designed to support primary care medicines optimisation [21]. Previous studies have not explored such a broad EMOS and how its utilisation may depend on a range of social and organisational factors. With that in mind, we aimed to examine how an EMOS was used for medicines safety activities in a primary care setting.

## Methods

### Study design and setting

Our research used a qualitative case study design, informed by the realist evaluation methodology (explained later). The study case was a clinical commissioning group in the South of England. In the English National Health Service (NHS), a Clinical Commissioning Group (CCG) is a clinically-led statutory NHS body responsible for the planning and commissioning of health care services for their local area, and cover a group of general practices within a local area. Whilst CCGs are made up of member practices they operate centrally providing support and governance to general practices that provide primary medical care as independent contractors. Within many CCGs a group of pharmacists, managers and other healthcare professionals are centrally placed and work as a medicines management team to optimise the quality and safety of prescribed medicines.

The CCG that forms our study was chosen because it was an early adopter of Eclipse Live, [21] the EMOS examined in this study, with all general practices signed up to the system. All practices used the same GP clinical IT system (In Practice Systems Vision). The sampling frame for data collection was stakeholders within the CCG’s geographical area who were using the system or potential users who were aware of it. This included doctors, pharmacists, healthcare managers and patients.

The EMOS examined in this study comprises a web-based user interface which securely extracts patient data from general practice patient records. Accessed separately from the general practitioners’ clinical systems, it allows different stakeholders access to real time anonymized patient data including medical histories of diagnoses, prescribed medications and test results. The use of the EMOS is intended to facilitate clinical audits of prescribing activity to identify patients at risk of ADEs, such as those receiving inappropriate combinations of drugs or not appropriately monitored. Patients can access the system through a “Patient Passport” that allows them to securely log on, view their medications and view and upload test results [21]. The EMOS allows clinicians to audit prescribing activity across a health care organisation and make comparisons against national guidelines.

### Recruitment and data collection

Individual participants were recruited on a purposive basis via the CCG or through community pharmacy networks, to represent the different stakeholder groups (see Table 1). Potential participants were contacted by telephone or email. To assist with recruitment and to allow the research team to obtain an initial understanding of the use of the EMOS informal discussions took place with users at two separate CCG sites; the study site and a CCG in the North of England. Five semi-structured interviews (lasting between 20 and 50 min) were conducted with three GPs and two CCG pharmacists, who were known to be using the system, between August and December 2014. Four homogeneous focus groups (lasting between 57 and 112 min) were conducted between September and December 2014, each with a specific group of stakeholders: GPs (2); community pharmacists (4); patients (4); and general practice managers (4). In the interviews and focus groups we explored experiences of working with the EMOS, perceptions of the system, benefits and drawbacks, the organisational structures and roles required for its use and the circumstances under which it was considered most effective. Data collection continued until saturation was reached and no new themes emerged from the interviews and focus groups.

**Table 1:** Participants and recruitment

Participants	Role	Use of EMOS
Interviews		
GP1-INT	General Practitioner	In general practice and as prescribing lead of Clinical Commissioning Group (CCG) medicines management team
GP2	General Practitioner	In general practice and as respiratory lead at CCG
GP3	General Practitioner	In general practice
CCGP1	CCG Pharmacist	Medication reviews in care homes
CCGP2	CCG Pharmacist	CCG medicines management team
Focus group A - General Practitioners		
GP4	General Practitioner	In general practice
GP1-FG	General Practitioner	In practice and as prescribing lead of CCG medicines management team
Focus group B – Community Pharmacists		
CP1	Community Pharmacist	Aware of, but no access
CP2	Community Pharmacist	Aware of, but no access
CP3	Community Pharmacist	Aware of, but no access
CP4	Community Pharmacist	Aware of, but no access
Focus Group C – Patients		
Pt1	Patient	Access through patient passport
Pt2	Patient	Access through patient passport
Pt3	Patient	Access through patient passport
Pt4	Patient	Access through patient passport
Focus Group D - General practice managers		
GPM1	General Practice Manager	In general practice
GPM2	General Practice Manager	In general practice
GPM3	General Practice Manager	In general practice
GPM4	General Practice Manager	In general practice
Observation		
CCGP1	CCG Pharmacist	Medication reviews in care homes

A number of possible participants were approached but declined to participate. Predominantly this was for reasons of time, workload or lack of use of the system. These included 2 pharmacist technicians, 2 GPs, 2 community pharmacists and 8 general practice managers

In addition, one observation was conducted of the system being used in practice (December 2014). Researcher MJ observed a CCG pharmacist at her usual workplace for a two-hour period, during which the pharmacist used the EMOS to conduct medication reviews of elderly care home patients. Field notes from the observation were added to the data set. The interviews, focus groups and observation were carried out by a male researcher trained and experienced in qualitative health research (MJ). The focus groups were co-facilitated by a female research pharmacist experienced in qualitative methodology (RLH). The researchers were not known to the participants prior to the study. Four interviews were conducted by telephone and one at the CCG offices, the focus groups were conducted at the CCG offices or at a local hotel. All participants gave written informed consent to take part in the study, and for the interviews and focus groups to be audio recorded and transcribed verbatim. Ethical approval for the study was granted by the NHS National Research Ethics Service (reference 14/NW/0113).

### Methodological approach: realist evaluation

**Fig. 1:**
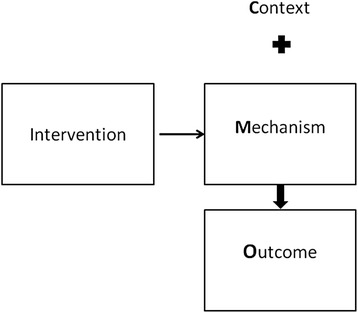
Realist Evaluation: Context Mechanism Outcome Configurations. Context: Pre-existing organisational, social or cultural circumstances. Intervention: Implemented into specific context. Mechanism: Specific and particular responses from human actors to the delivery of the intervention. Outcomes: product of mechanisms activated within the specific context

Complex interventions, such as those involving the implementation of healthcare IT, can be understood from a “realist evaluation” perspective which seeks to explain the ways the intervention might work, for whom and under what circumstances [22, 23]. Realist evaluation draws from realist philosophy in which human action is seen as occurring within different layers of social reality. Actions only make sense if they are considered as part of this social reality with its associated rules, social norms and regulations [23]. Realist evaluation asserts that a set of outcomes is the product of particular responses from human and technological actors within the system (“mechanisms”). These mechanisms are activated in a given set of organisational or social circumstances (“context”) [23, 24]. A combination of contexts and the associated mechanisms leads to outcome(s) for a given intervention [23]. Given the complexity of healthcare interventions, [25–27] realist evaluation provides a detailed understanding of what makes an intervention work, rather than a simple cause-and-effect relationship between an intervention and its outcome(s). The latter can indicate whether or not an intervention has worked, but provides limited insights into how or why the identified outcomes were obtained [23, 24]. Realist evaluation presents these findings as a set of links between contexts, mechanisms and outcomes (so-called “CMO configurations”) (Fig. 1) [22–24, 28–30].

### Analysis

Consistent with qualitative realist evaluation the analysis was cumulative and iterative [22–24, 28–30]. The data were analysed using a thematic approach, with each theme representing a set of CMO configurations (CMOs). Similar to previous realist evaluations [24, 31] an a priori set of CMOs was developed deductively from available literature [12–15, 19, 32] and informal discussions with users of the EMOS. These included ways in which the intervention led to changes in work practices [12], changes to the flow of information [13, 32] and the goals of the system. These provided the initial thematic framework for data analysis. Early findings were discussed in subsequent focus groups in an iterative approach consistent with realist evaluation [23]. Transcripts of the interviews and focus groups were read and discussed across the research team. A set of thematic codes, based on the initial framework, was applied to the transcripts using the QSR Nvivo 10 application to organise the data. These codes identified potential outcomes of the intervention. The outcomes were then grouped under new themes that emerged from the data. Finally, having determined what the outcomes were, we interrogated the data further for the mechanisms and contexts that might have led to them. Hence, we generated CMO configurations from the data which in turn were further organised thematically into the three groups detailed in the results below. The data coding and analysis were led by MJ, with regular discussions about codes and emerging themes and CMO configurations held with all co-authors, including a patient representative.

## Results

Consistent with a realist evaluation the findings were conceptualised as CMO configurations; the circumstances and ways in which the EMOS was used were perceived to lead to a number of medication safety outcomes. These CMOs were organised into three groups based upon the ways the system was utilised: access; engagement or disengagement with the system; the monitoring of prescribing; and work practices. Within each group we identified mechanisms, and contexts within which these mechanisms were activated, that led to given medication safety outcomes such as patients’ electronic health records being screened to identify potentially hazardous prescribing events.

### Engagement and disengagement

The first group of CMOs concerned access, engagement and disengagement (Tables 2 and 3). In the first of these CMOs, the EMOS focused healthcare users’ attention on medication rather than on disease.

**Table 2:** Context-Mechanism-Outcome configurations concerning access and engagement

Context	Mechanism	Outcome
General Practitioner monitoring individual patients	Focuses attention on medications	Attention focused on patients most in need of review
General Practitioner prescribing audited and monitored in practices	Proactively conducting own audits	Practice prescribing patterns benchmarked against each other across the Clinical Commissioning Group
Communication between Clinical Commissioning Group and General Practitioner	Real time feedback	Patients reviewed to ensure appropriate monitoring, to optimise medications, or to avoid dangerous combinations of drugs
Clinical Commissioning Group conducting searches of prescribing based upon “projects” and “initiatives”		Prescribing patterns and trends benchmarked against national targets and guidelines
Clinical Commissioning Group encouraging clinicians to be engaged in more proactive safety management	Engagement of practices in using the system for feedback Voluntary engagement by clinicians Audits conducted as a means of support to General Practitioners	The effectiveness of safety initiatives audited more quickly Improved engagement with safety monitoring of prescribing

**Table 3:** Context-Mechanism-Outcome configurations concerning disengagement

Context	Blocking Mechanism	Outcome not achieved
Communication between Clinical Commissioning Group and general practitioners	Feedback on alerts requires logging in	Potential delays in patients being reviewed
	Reliance on alerts being sent out centrally	
Information technology use in General Practice	Lack of use/not logging in to the system	Potential delays in review of patients
Community pharmacists conducting medicine use reviews with patients	No access to additional information	Opportunity for more appropriate and directed medication review lost
Community Pharmacy	Perceived conflict and lack of ownership	Limits potential improvements in quality of care for patients
Patients using the electronic medicines optimisation system	Facilitated use by healthcare professional	Lack of direct access to information to benefit shared care and self-management
	Difficulties obtaining passwords and logging on

*“Say you are monitoring renal function and you look and the eGFR [patient’s filtration rate] has gone down to 29 and it was 31 the month before. You’re thinking, well that’s okay, we’ll just monitor that, you fail sometimes, […] one fails to think, ah, I need to review the allopurinol, I need to renew the metformin, because it is so, so, easy to focus on a disease and that’s, I think, where Eclipse can come in.* (GP1-INT)

Engagement with the system by GPs could therefore lead to more focused patient reviews. The system could be used for feedback, giving them “*some idea as to who’s perhaps even more engaged than others”* (CCGP2). If activated, this mechanism could “*inform the CCG about how well safety initiatives are happening”* (CCGP2) and lead to a speedier audit and feedback of safety initiatives rolled out centrally.

Increased engagement with safer prescribing could be sustained by voluntary engagement with the EMOS on the part of the practices; this was said to reduce a *“big-brother”* (GP1-INT) relationship with the CCG, challenge the belief that it was a tool primarily for the CCG pharmacists, and give a greater sense of ownership of the system within general practice. However, GPs could instead end up relying upon the medicines management team to send out alerts, disengaging them from proactively using the system and reinforcing CCG ownership. Engagement was to be encouraged financially in the future by building a requirement to use the EMOS into the *“prescribing incentive scheme”* (CCGP2). In contrast, engagement was discouraged by blocking mechanisms in the context of IT use in general practice. One GP (GP2) stated that they and only one other colleague used the EMOS. Such task allocation meant that within their practice they operated as a prescribing lead where they took responsibility for auditing and monitoring the prescribing within their practice, and therefore were the only ones expected to use the system. Another GP remarked, in terms of seeing alerts in the system, *“I don’t commonly open the software full stop”* (GP4) a barrier that was related to time pressures:-

*“a third of my time (is) seeing patients, two-thirds of my time doing paperwork and an extra mystical 10 or 20% of time […] Eclipse fits into that last 10, 20% of time that doesn’t really exist.”* (GP4)

Other stakeholders were also disengaged from the system. Community pharmacists were aware of it and perceived potential benefits involving increased information through access to care records that could inform medicines use reviews, improve communication with GPs and *“influence a decision to sell a medicine or supply a medicine.”* (CP3). However, they had not been given access by the CCG, nor had access through the “Patient Passport”, though such access had been planned. This was attributed to perceived difficulties with sharing information, issues of confidentiality and a perception that *“GPs often see themselves as the custodian of the patient record”* (CP3) which meant *“historically a barrier to sharing that information”* (CP3). Community pharmacists had been involved in patient passport initiatives that could have given them access to the EMOS but issues of confidentiality, delays and poor communication with the CCG and general practices had led to them being denied access.

A limited number of patients had access to the EMOS through the patient passport. They saw this as potentially valuable in giving access to information about medications and their conditions, which would in turn have a positive bearing on self-management and shared care. However, this was prevented by a blocking mechanisms concerning access, *“The first problem I had was I couldn’t log in at all”* (P2). Patients also felt that they would get best use out of the system if this was facilitated and interpreted by a health professional.

*“I think that’s why it’s important to, it’s not just to be used on its own, it’s to be used with, to be used with a clinician of some kind to actually help you to interpret some of that stuff, because some of it is, I mean when you look at high haemoglobin levels or the glucose levels, […] Which are the bad ones? Which is this? What does this mean? “(*P1)

### The monitoring of prescribing

The monitoring of prescribing across general practices (see Table 4) was undertaken by pharmacists and GPs placed centrally at the CCG.

**Table 4:** Context-Mechanism-Outcome configurations concerning the monitoring of prescribing

Context	Mechanism	Outcome
Clinical Commissioning Group engagement with prescribing alerts	Alerts designed and results fowarded to practices	Prescribing patterns and trends benchmarked against national targets and guidelines
	Identify specific patients	Pre-emptive or timely review of individual patients
Monitoring prescribing by conducting searches based upon local “initiatives”	Efficient use of time	Prescribing patterns benchmarked across the Clinical Commissioning Group
	Highlight suboptimal prescribing	Reduction of knowledge gaps to optimise use of medicines
	Reward good practice	

We identified two contexts within which mechanisms were activated. The first of these concerned the engagement with prescribing alerts issued by the CCG. Alerts that related to the implementation of national guidance were designed and disseminated to general practices. These allowed for bespoke searches of prescribing data to be run across all general practices within the CCG. This in turn allowed for benchmarking against criteria set by national guidelines. One respondent (GP1-INT) acknowledged that the existing alerts embedded within the system could be used, but that they were unwieldy because of their large number so were not commonly used. Similarly, one CCG Pharmacist (CCGP2) said there was a lack of confidence in these alerts, because of a lack of knowledge about the content of the underlying algorithms used to generate the existing alerts, so they were seldom used. The engagement with prescribing alerts also allowed the activation of a mechanism for identifying specific patients, which was seen as more likely to lead to a timely review of patients.

*“You [can] pin [the alert] to [specific patients]. So if you say […] metformin shouldn’t be prescribed with an eGFR less than 30 and these are the patients who you need to consider in this category it’s such a more meaningful event.”* (GP1-INT)

The second context concerned the CCG setting up their own searches based upon local initiatives. Within this context one mechanism allowed for searches to be conducted speedily across all practices within the CCG. This was a change in working, where in the past *“trawling round all […] practices”* (CCGP2) had *“[taken] us about three to four weeks”* (GP1-INT). Since the introduction of the intervention, *“we ran the same search and literally […] 90 min without actually leaving your desk, you’ve got the results”* (GP1-INT). Using the system helped to identify prescribing patterns and *“to have the ability to look at the prescribing by practice […] so we could compare […] the prescribing of a drug one practice to another”* (GP1-INT). Participants saw this as leading to prescribing patterns being benchmarked across the CCG. Additionally, the EMOS was seen as an educational tool that could reduce knowledge gaps and change prescribing behaviour by highlighting suboptimal prescribing within and across practices *“because we could identify those patients receiving whatever strength, notify GP within the system and […] got 100% adherence to this safety thing”*(GP1-INT). This educational outcome was further enhanced by rewarding good practice: *“if there are some practices that are demonstrating very good prescribing, then we’ve picked those out as well and highlighted those”* (CCGP2).

### Work practices

The final group of CMOs concerned the effect of the EMOS on work practices (Table 5). This involved a number of different stakeholders in general practices: GPs; practice managers; and practice-based pharmacists.

**Table 5:** Context-Mechanism-Outcome configurations concerning work practices

Context	Mechanism	Outcome
Multiple administrative work practices	Logging on, responding to alert, and reviewing patients *through* the system	Patients reviewed to ensure appropriate monitoring, to optimise medications, or to avoid hazardous combinations of drugs
Pre-existing division of labour within General Practices	Task allocation	
General Practice workload	Task Prioritisation	Pre-emptive or timely review of individual patient
Pharmacist workload	Existing work practices developed and adapted	Can result in a more focused medication review
Pharmacist undertaking reviews in care homes	Accessing easily readable and informative data	
	Necessary workarounds to overcome technical issues	
	Necessary workarounds to find patient details	

The first context here concerned administrative work practices. Some practices relied on alerts being sent to them by email rather than proactively seeking the alerts by logging on to the EMOS. The process of responding to alerts varied, but often involved transferring information from email to paper in addition to logging on to the system, causing a delay.

*“The alert is printed off on a piece of paper which [then] sits in my in tray with 500 other items of equal urgency, and […] it might be that I have to work my way down through that pile over a period of a few months.”* (GP4)

Reviewing the patient through the system was a more successful mechanism that gave immediate feedback to the CCG, avoided the delays, and provided clear and speedily accessible information in a readable form where: *“you can plot the graphs [and] quickly eyeball 100 patients in a couple of minutes.”* (GP1-INT)

Within the context of pre-existing divisions of labour within practices, EMOS was seen to require a specific task allocation which would be *“certainly led by a clinician and most likely performed by a clinician”* (GP4). There was variation in the ways the EMOS was used by either practice managers or GPs. One practice manager said that once an alert was received they took responsibility for it:

*“I pass it on to the GP and get them to respond to me, and then I update Eclipse […] the doctor’s don’t access it at all”* (GPM2)

Whereas in another practice the responsibility for accessing the system was the GP’s:

***“*** *The GP actions it, I don’t have any more responsibility for it after that […] They go into Eclipse, they do it, […] I had to remind one GP today, I just wanted to check they had actually reviewed this patient”* (GPM1)

If the system was used effectively then patients would be reviewed but, as noted by the general practice manager above, it was possible that the task allocation could act as a blocking mechanism (that is, inhibiting the effect of the system) if GPs had to be reminded to review patients.

Within the GP workload context, mechanisms associated with task prioritisation could lead to the timely review of patients. To utilise the system effectively, GPs had to juggle competing tasks and prioritise. If GPs were *“getting pertinent alerts that they feel are relevant”* these alerts were seen with *“virtually no negativity.”* (GP1-FG)

For pharmacists undertaking medication reviews in care homes, the system saved time by giving more speedy access to information, *“there and then in front of you”* (CCGP1) allowing for a more focused review. The system gave the pharmacist the opportunity to send recommendations to the GP based on information about medications, test results, conditions and demographic factors. This information was easily accessed through the EMOS and findings easily interpreted.

*“The benefit of Eclipse is you can log on and look at the graph and you can see the basic trend of blood pressure, of cholesterol, of weight et cetera, on a beautiful graph which is so easy to read with the red/amber/green bits, it’s so clear what’s going on.” (*CCGP1)

Effective use of the system required some adaptations and improvisation on the part of the users. For example participant CCGP1 whilst carrying out tasks in a care home, had to adapt ways of obtaining passwords for the system to deal with limited internet access. Pharmacists *“beforehand were trying to look up all the stuff on Eclipse whilst we were in the care home”* (CCGP1) but had adapted their activities in order to have *“more information to start off with (and) use Eclipse for less time in the care home, but in a more directed manner”* (CCGP1). Limitations to the information available in the system, necessitated workarounds in order to obtain further patient details; *“because it doesn’t list actual allergies”* (CCGP1) and *“we can’t look at letters”* (CCGP1). This meant finding out more information from the general practices before the visit to the care home or returning to general practices to obtain *“any relevant letters from consultants or anything like that”* (CCGP1).

## Discussion

Our study has identified variations in stakeholders’ experiences of the IT intervention across primary care, which potentially affects its successful implementation. The capacity to audit prescribing across practices allowed for the practices to be benchmarked. One particular benefit of the EMOS is the ability to swiftly review specific patients and groups of patients to ensure they have appropriate monitoring, to optimise dosages or to avoid hazardous combinations of medicines, which may result in safer prescribing. The system was valued by the clinicians and pharmacists placed centrally at the CCG because it could be utilised to access prescribing information and could lead to the timely review of patients at risk of adverse drug events. There was a sense that the system was for the CCG and owned by them. This created barriers to use elsewhere. Centrally the CCG encouraged access to general practices but could limit the engagement for others. There was therefore a “top down” implementation that was dependent upon soft governance from the CCG in the form of incentives and permission for access. Elsewhere, the value of the EMOS was dependent upon two factors: the flow of information and work practices.

Consistent with previous literature, participants in our study had adapted work practices to use the EMOS [13, 19]. Whilst prioritising tasks and enacting workarounds contributed to effective use of the system, some work practices acted as blocking mechanisms; for instance, underutilising the system by making paper copies of alerts that were designed to be read and responded to on screen. In a study of GP practices’ handling of secondary care information, [33] delays were seen to be caused by similar sub-optimal work practices.

It was perceived that the flow of information between the CCG and individual practices facilitated engagement with the system. Practices were more engaged when alerts were limited, more relevant and the system was trimmed down to local alerts based on local projects rather than using a whole catalogue of embedded alerts within the system. Consistent with previous research this tailoring of alerts allowed for time saving, avoided alert fatigue [12] and so encouraged greater engagement. Ojeleye and colleagues [34] have likewise found that the tailoring of alerts maximised the likelihood of action being taken. Where practices logged in to the EMOS to review patients they provided feedback to the CCG that they were proactively engaging with the system. In our study this engagement was said to have an educational impact similar to previous findings [35].

### Strengths and weaknesses

The particular strength of this study is the novel use of a realist evaluation approach to examine an information technology intervention in primary care. This allowed us to explore in detail the ways the EMOS was used and the potential effect this had on medication safety outcomes. Recent guidelines advise that evaluation should examine in detail how the intervention works and the interactions of different stakeholders [36].

A potential limitation of our study was that it focused upon one single CCG. Whilst there was inclusion of all relevant stakeholders the number of participants in each group was small given the size of the case study. The CCG had been an early adopter of this EMOS but it was used less widely than anticipated. This limited our understanding of how the system could be used by the widest range of stakeholders and in different contexts within primary care particularly since the use in community pharmacy and among patients was limited. Due to the nature of our study design, we were reliant upon respondents’ subjective accounts and were unable to assess the medication safety outcomes directly.

### Implications for medicines management and further research

Consistent with other literature around the implementation of IT systems [13, 17, 37, 38], our findings show how disengagement from the system undermined its effectiveness. Sociotechnical approaches have understood the implementation of IT in terms of an interaction between the technology and the human. Our findings highlight implications for the implementation of other IT systems in medicines management, and more broadly in healthcare, and that they may be understood from a sociotechnical view.

Specifically, we found that greater ownership of the system across the workforce, and more embeddedness within existing work practices [37], could lead to better utilisation across primary care, with potential benefits for medication safety. The use of the system was undermined by a perception amongst several stakeholders that the EMOS was owned by the CCG. Partly this perception was the design of the system as a tool to be used for audit and feedback centrally. The value and potential for the system to be used locally, was undermined by the ownership of it by the CCG and by the understanding that it was a population level audit tool. Utilisation was further undermined by time pressures in general practice, a lack of access to and a lack of knowledge and awareness of the potential benefits of utilising the EMOS. Participants in our study speculated that general practices more engaged in the use of the system might have dedicated prescribing leads who are more likely to run their own audits and as such were more proactive in managing medication risks. The lack of involvement from a broader range of stakeholders, including community pharmacists and patients, prevented the exploitation of potential benefits of the EMOS in enhancing shared care, self-management and medication reviews; all of which could have an impact upon medication safety.

Our realist evaluation was a case study was of a single CCG. We suggest that the CMO configurations we conceptualized here would be valuably applied to further evaluation of this system in use in other CCG areas, in a process of cumulation and further theory testing [22]. This would build upon how the system could be further implemented. Furthermore our study CCG was a predominantly rural area with a relatively small number of general practices and all practices used the same clinical system. Further research on the use of this EMOS in other CCGs, where different clinical systems are used, might lead to different contexts being identified; for example, busier urban practices might have high staff turnover, which creates greater variations in prescribing behaviour and user interaction [12]. A realist evaluation of this EMOS might also be run in a longitudinal manner, alongside the implementation of the intervention. This would help to track changes to work practices as the intervention was embedded into existing work behaviour [39, 40].

There are wider implications for the evaluation and implementation of other IT systems in primary care. In our realist study, contexts were not only time and space but also pre-existing work practices, workload and divisions of labour. IT systems in healthcare are often interrelated, and their interactions with users vary and evolve over time [41]. This creates complexity in the way that healthcare organisations operate [15, 32]. Future evaluations of healthcare IT systems could look to realist evaluation as a way of unpicking this complexity in order to optimise their use.

## Conclusions

The implementation and adoption of an electronic medicines optimisation system may improve medication safety in primary care settings by identifying those patients at risk of an adverse drug event. However the use of such a system was found to be dependent upon adapting work practices and undermined by perceptions of ownership, lack of access, lack of knowledge and awareness, and expectations concerning variable task allocation. As a result some stakeholders had limited or no engagement with the system.

To fully realise the potential benefits for medication safety of the EMOS, there needs to be better utilisation across primary care and with a wider range of stakeholders. Future roll out of this system might consider how perceptions of ownership might impact upon utilisation. Engaging with all potential stakeholders and users prior to implementation might not only allay a sense that the system is owned centrally but increase knowledge and awareness of the potential benefits of the system. We found that realistic evaluation was a valuable approach to unpick the complexity of an IT intervention in primary care and would recommend that this approach is adopted for similar evaluations.

## Abbreviations

ADE: Adverse drug event
CCG: Clinical commissioning group
CMO: Context mechanism outcome (configuration)
EMOS: Electronic medicines optimisation system
GP: General practitioner
IT: Information technology
